# Successful treatment of massive biventricular thrombi associated with myocarditis: a case report

**DOI:** 10.3389/fcvm.2025.1530548

**Published:** 2025-04-28

**Authors:** Chunshui Liang, Hong Liu, Zhezhe Cao, Tianbo Li, Yong Wang, Yingbin Xiao, Ruiyan Ma

**Affiliations:** Department of Cardiovascular Surgery, Xinqiao Hospital, The Army Medical University, Chongqing, China

**Keywords:** biventricular thrombi, myocarditis, cerebral infarction, cerebral hemorrhage, anticoagulation

## Abstract

**Background:**

Massive biventricular thrombi associated with myocarditis are rare and pose significant management challenges.

**Case presentation:**

A 24-year-old male from a plateau region presented with dyspnea, chest pain, and cerebral infarction/hemorrhage. Imaging revealed giant biventricular thrombi (left: 81 × 62 × 59 mm; right: 59 × 16 mm) and LVEF of 25%.

**Management:**

Multidisciplinary therapy included anticoagulation and GDMT.

**Outcomes:**

Complete thrombus resolution occurred by 10 weeks, with improved cardiac function (LVEF 48%) and no recurrent embolism.

**Conclusion:**

Drug therapy may be effective for massive biventricular thrombi in myocarditis.

## Introduction

Ventricular thrombi are rare but carry a high risk of systemic embolism and mortality (1). Myocarditis, characterized by inflammatory infiltration of the myocardium, may lead to endothelial injury, blood stasis due to impaired ventricular function, and a hypercoagulable state—factors that align with Virchow's triad and predispose patients to thrombus formation (2). The resolution of small mural (laminated) ventricular thrombus is predominantly accomplished through anticoagulation (2), however, the management of large ventricular thrombus remains uncertain, with only a limited number of cases documented in the literature (3–11). This case represents the largest reported instance of biventricular thrombi, complicated by cerebral infarction and hemorrhage, and the treatment process was notably complex. This case aims to demonstrate the feasibility of medical therapy for massive biventricular thrombi in myocarditis, particularly in patients with concurrent cerebral infarction and hemorrhage, and to highlight the challenges in balancing anticoagulation risks.

## Case presentation

A 24-year-old male resident of the plateau region presented to the emergency department with a 2-day history of weakness and anorexia, following a month-long period of dyspnea, chest pain, and dry cough. The patient was conscious and exhibited stable vital signs, including a temperature of 36.6℃, a pulse rate of 100 bpm, a respiratory rate of 18 bpm, and a blood pressure of 152/84 mmHg. The physical examination revealed an arrhythmia (ventricular premature beats) without associated murmurs, and the presence of lower limb edema, but no jugular vein distension. The patient had a history of smoking 1 pack a day for 4 years and ceased smoking 7 months prior.

**Figure 1:**
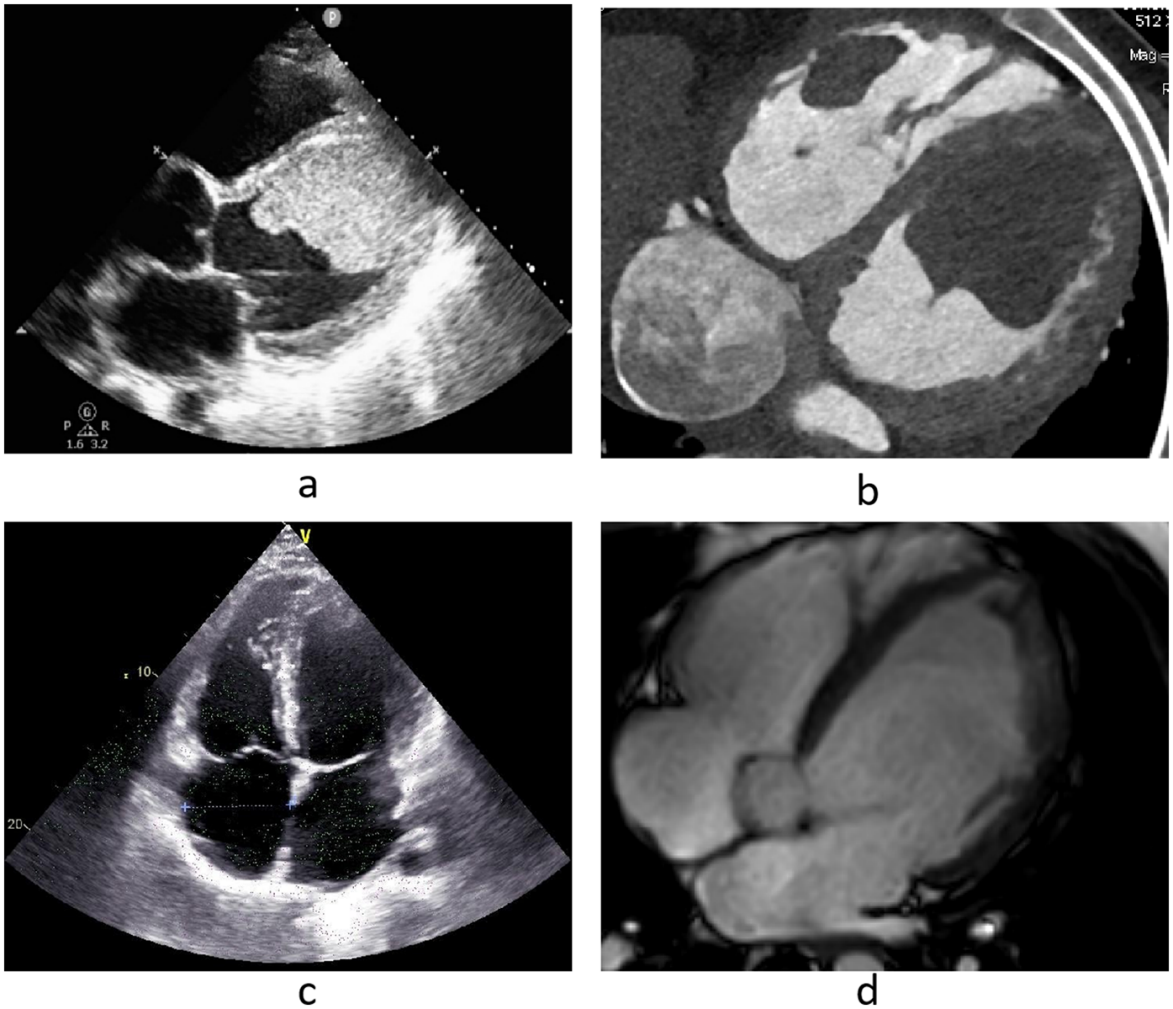
**(a,b)** Echocardiography and cardiac CTA on admission, **(c,d)** echocardiography and cardiac MRI before discharge.

**Figure 2:**
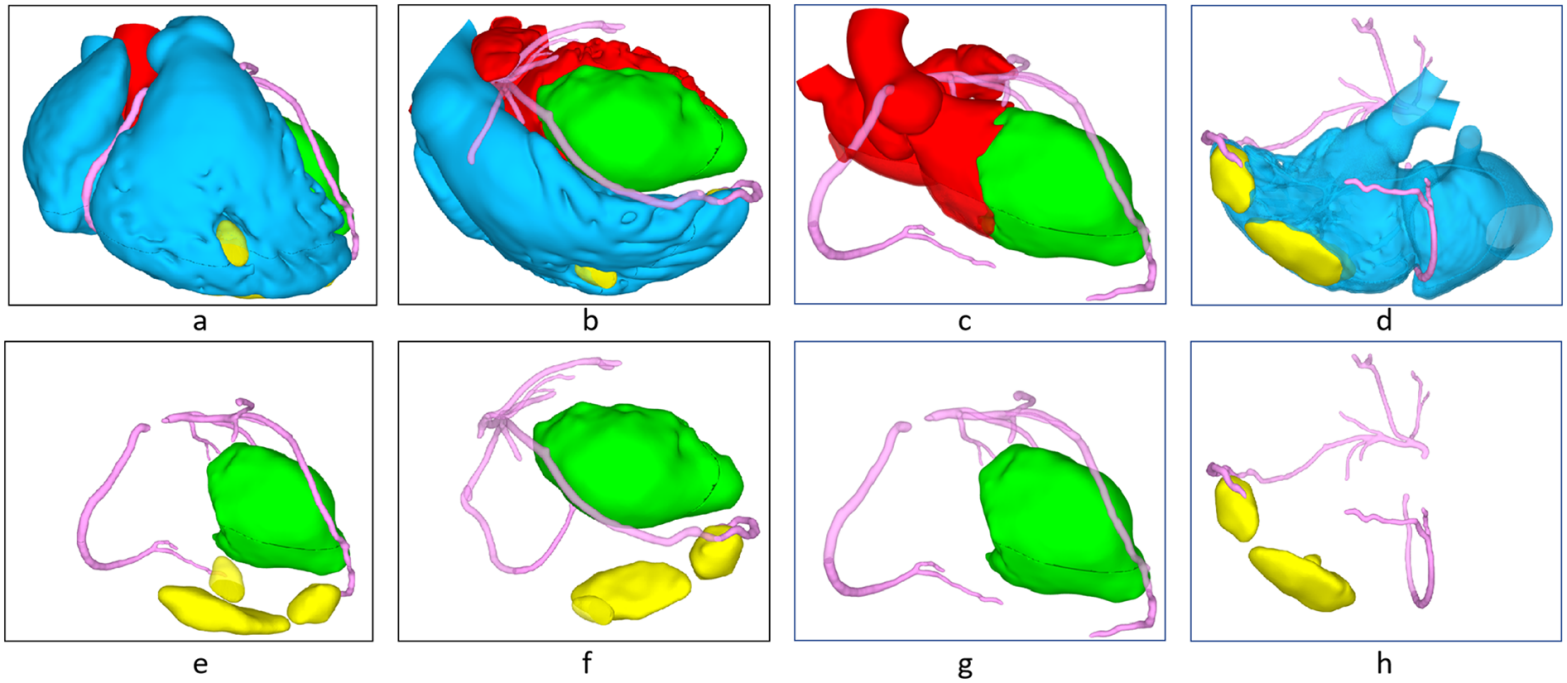
3D reconstructed image (red: left blood flow, blue: right blood flow, green: left ventricular thrombosis, yellow: right ventricular thrombosis), **(a,e)** frontal view, **(b,f)** bertical interventricular septal plane, **(c,g)** left ventricualr outflow tract plane **(d,h)** right ventricular outflow tract plane.

Laboratory test revealed a hemoglobin of 187 g/L, a hematocrit of 56.4%, a erythrocytes count number of 6.67 × 10^12^/L, a positive troponin assay, a C-reactive protein (CRP) level of 68.62 mg/L, a procalcitonin of 0.34 ng/ml, an albumin level of 22.3 g/L, and a B-type natriuretic peptide (BNP) of 1,370 pg/ml. Thromboelastography, coagulation tests, tumor markers test, immune-related test (including plasma protein S, plasma protein C, anticoagulant antibody spectrum, anti-cardiolipin antibody, anti-streptolysin O) were within normal. Thrombophilia tests were not performed due to conditions being limited. A Electrocardiograph indicated ST segment changes and ventricular premature beats. A transthoracic echocardiography (TTE) revealed a anteroposterior diameter of 62 mm for the left ventricle and 55 mm for the right ventricle. The examination revealed that the tricuspid valve regurgitation was moderate, with a regurgitation velocity of 237 cm/s and a peak pressure gradient of 22 mmHg. The left ventricle ejection fraction (LVEF) was measured at 25%. The tricuspid annular plane systolic excursion (TAPSE) was measured to be 12 mm. Additionally, the presence of a potential biventricular thrombi was detected (left ventricular thrombus dimensions: 81 mm × 62 mm × 59 mm; right ventricular thrombus dimensions: 59 mm × 16 mm) (Figure 1a). Cardiac computed tomography angiography (CTA) revealed a low-density shadow indicative of a biventricular mass (Figure 1b), raising the possibility of thrombus. No abnormalities were detected in the coronary arteries, pulmonary artery, or thoracic aorta. Additionally, severe pathology was noted in both lungs, accompanied by a small amount of fluid in the bilateral pleural cavities. 3D models are used to better understand ventricular thrombi (Figure 2). Positron Emission Tomography-Computed Tomography (PET-CT) scan revealed low-density shadows in both the left and right ventricles, with no abnormal increase in FDG metabolism. Multiple acute infarction were observed in the bilateral cerebellar hemispheres, alongside slightly high-density shadows indicative of infarction and hemorrhage. Pulmonary plaque shadows were noted, accompanied by increased FDG metabolism suggestive of inflammation. Additionally, diffuse pericardial FDG metabolism was observed, pointing to inflammatory lesions. Multiple mucosal effusions and systemic subcutaneous edema were also considered in the findings. The magnetic resonance imaging (MRI) myocardial perfusion imaging revealed the left and right ventricular filling defects, the potential presence of thrombus, the left ventricular wall and subendocardial late gadolinium enhancement (LGE), the possibility of myocarditis or cardiomyopathy, the reduced systolic function of the left ventricle with an LVEF of 17%, and the presence of a small amount of fluid in the pericardial and pleural cavities. Neuroimaging, including brain computed tomography (CT) and diffusion-weighted imaging (DWI), revealed multiple acute cerebral infarctions in the bilateral fronto-parietal and occipital lobes, the left thalamus, and the bilateral cerebellum, along with a small amount of hemorrhage in the right cerebellum. Cervical vascular ultrasound revealed the presence of mural thrombus in the right internal jugular vein catheter, measuring approximately 0.6 cm × 2.8 cm.

## Treatment strategy

Following a multidisciplinary consultation, drug therapy was recommended, however, patients may experience systemic embolism and pulmonary embolism in the course of treatment. While the patient was administered a low a dose of enoxaparin (40 mg BID) due to the presence of cerebral hemorrhage, metoprolol 23.75 mg QD, spironolactone 20 mg QD, dapagliflozin 10 mg QD, sacubitril/valsartan 24/26 mg BID for GDMT, along with other supportive treatments. The patient underwent daily monitoring through TTE. After a 1-week treatment period, TTE revealed an increase in LVEF to 33% and in TAPSE to 14 mm. The size of the biventricular thrombi remained unchanged. Cervical vascular ultrasonography revealed the presence of thrombus not only in the right internal jugular vein but also in the left subclavian vein and the proximal segment of the left internal jugular vein, with significant mobility observed. Following a multidisciplinary discussion, considering that the risk of cerebral hemorrhage has decreased, but the risk of thrombosis is still very high, the therapeutic dosage of enoxaparin was adjusted to 70 mg (1 mg/kg) Q12H. CRP and procalcitonin returned to normal 10 days after admission.

Two weeks post-admission, TTE revealed a reduction in the left ventricular thrombus to 62 mm × 60 mm × 41 mm and the right ventricular thrombus to 46 mm × 18 mm. Additionally, the LVEF measured of 36%, and TAPSE was at 15 mm. However, there was no alteration in ventricular size, and mobile was noted on the free surface of the left ventricular thrombus. Cardiac CT revealed the absence of pulmonary embolism, while brain CT revealed a reduction in the cerebral infarction and no evidence of new hemorrhage. The patient exhibited no new signs or symptoms. Considering the patient's history of cerebral hemorrhage extending beyond 2 weeks, the risk associated with extracorporeal circulation surgery was significantly diminished. A multidisciplinary consensus recommended prioritizing surgical thrombectomy due to the observed mobility of left ventricular thrombus. The complexity of thrombectomy procedures results in extended operation times. The prolonged duration of the surgery can further compromise cardiac function, markedly elevating the risk of postoperative low cardiac output syndrome and cerebral hemorrhage. Consequently, the likelihood of requiring postoperative intra-aortic balloon pumping (IABP) or extracorporeal membrane oxygenation (ECMO) is significantly increased, which in turn raises the risk of cerebrovascular events.

Two days subsequent to the decision to perform a thrombectomy, TTE revealed a reduction in the thrombus size within the left ventricle to 56 mm × 45 mm × 38 mm and within the right ventricle to 19 mm × 13 mm. Additionally, cervical vascular ultrasound revealed a decrease in the thrombus size within the right internal jugular vein. No thrombus was detected in the left subclavian vein or the left internal jugular vein. The patient exhibited no new clinical symptoms. Following a multidisciplinary consultation, it was determined that the thrombus had significantly decreased in size, thereby permitting the continuation of medication and a transition from enoxaparin to rivaroxaban for anticoagulation therapy. Nevertheless, the risk of embolization persisted.

Following sustained drug management, including anticoagulation and GDMT, cervical vascular ultrasound revealed complete resolution of the right internal jugular vein thrombus 3 weeks post-admission. Additionally, cardiac MRI revealed full resolution of the right ventricular thrombus at 4 weeks and the left ventricular thrombus at 10 weeks post-admission (Figure 1d). Brain CT revealed that the cerebral infarction reduced in size, with no indications of new cerebral infarctions or cerebral hemorrhages. Prior to discharge, TTE revealed normal size and function of the right ventricle, with a TAPSE of 20 mm. The anteroposterior diameter of the left ventricle measured at 68 mm, the LVEF was 48%, and no valvular regurgitation was detected, with a tricuspid regurgitation velocity of 346 cm/s and a peak pressure gradient of 48 mmHg. The patient did not present with any new symptoms of embolism, and there was a marked improvement in symptoms such as weakness, anorexia, dyspnea, chest pain and dry cough.

In the subsequent treatment regimen, the patient's dose of sacubitril/valsartan was adjusted to 36/39 mg BID, with no other drug modifications. The patient's vital signs included a resting heart rate of 70 BPM and a blood pressure of 100/70 mmHg. Given the patient's medical history of biventricular thrombus and myocarditis, it was recommended that the patient should avoid returning to work at high altitudes and continue rivaroxaban therapy for a duration of 6 months. In this cases of left heart-associated pulmonary hypertension, initial observation was conducted, and drug of pulmonary hypertension treatment was determined based on the results of subsequent patient evaluations.

## Follow-up

The patients were followed up for 2 weeks, 1 month, 3 month and 6 month, and no new clinical symptoms appeared, resting heart rate was 65 BPM, blood pressure was 101/65 mmHg. TTE revealed the LVEF was 44%, and no valvular regurgitation was detected, with a tricuspid regurgitation velocity of 251 cm/s and a peak pressure gradient of 25 mmHg. Cardiac MRI showed no ventricular thrombus. The patient returned to daily life, but did not perform physical work.

## Discussion

According to Virchow's triad, the pathogenesis of left ventricular thrombus involves the interplay of three critical factors: (1) blood stasis resulting from diminished ventricular function, (2) endocardial injury, (3) a state of inflammation or hypercoagulability. The relative contributions of these factors to the formation of LV thrombus depend on the etiology of myocardial dysfunction and its duration. While regional endocardial injury and inflammation may play a predominant role in an acute myocardial infarction (MI), stasis resulting from diminished LV function is likely to be the principal factor in dilated cardiomyopathy (DCM) (2). The patient in this case was considered myocarditis: did not have instrumental lesions, obvious clinical symptoms, troponin was positive, ST segment changed in ECG, and serious diminished in ventricular systolic function. He experienced persistent dyspnea and pain over a 1-month period, suggesting the presence of heart failure during that time. Despite the patient's young age and good physical condition, which allowed for tolerance of prolonged impaired ventricular systolic function, the myocarditis had also resulted in damage to the endocardial membrane. The risk of hypercoagulability is heightened in patients residing at high altitudes due to elevated hemoglobin levels. Three factors facilitate the development of biventricular thrombi in such patients. The adhesion of thrombus to the ventricular wall further impairs ventricular muscle contraction, exacerbating symptoms of nausea and weakness. This clinical presentation is indicative of recent thrombus formation. LV thrombus is reportedly less prevalent in DCM compared to ischemic cardiomyopathy. This discrepancy may be attributed to underdetection, as the incidence rates of thromboembolic events have been observed to be comparable between the two conditions (12). Limited data also suggest that protuberant thrombi may resolve earlier than mural thrombi [OR, 3.2 (95% CI, 1.1–8.89); *P* = 0.026] (13), which may affect long-term thromboembolic potential.

In our case, the complete dissolution of the right ventricular thrombus was observed within 4 weeks, and the left ventricular thrombus required 6 weeks. At present, giant ventricular thrombus is predominantly documented through case reports (3–11), with treatment strategies including anticoagulation, fibrinolysis, and surgical thrombectomy. Nevertheless, the lack of substantial evidence-based medical data has resulted in persistent debates concerning the most effective therapeutic approach (2). For patients demonstrating adequate cardiac function, surgical thrombectomy is typically recommended (9, 10). In instances of ischemic cardiomyopathy, where cardiac function is compromised, a combination of bypass surgery and thrombectomy is regarded as an efficacious treatment strategy (3). Conversely, for patients with severely impaired cardiac function, thrombolysis is the preferred option among most clinicians (4–8, 11). It is imperative to evaluate the risk of thrombus embolization and to make decisions through multidisciplinary discussions. In addition to ventricular thrombus concomitant with myocarditis, our patient presented with both cerebral infarction and hemorrhage, cerebral infarction is most likely caused by the loss of ventricular thrombus, followed by cerebral hemorrhage after infarction. Thereby markedly complicating the management of anticoagulation therapy and surgical intervention. Balancing the benefits and risks of these therapeutic approaches proved to be exceedingly challenging and complex. When low doses of enoxaparin were given in the early stage to prevent cerebral hemorrhage, the patient's ventricular thrombus did not change, and further venous thrombus occurred. After multidisciplinary discussion, the biventricular thrombi was gradually dissolved after adjusting the enoxaparin dose to normal. However, partial dissolution of ventricular thrombus also leads to thrombus mobile, while GDMT treatment gradually restores the contractility of the left ventricle, which greatly increases the risk of ventricular thrombus drop out. Therefore, it is necessary to closely observe the symptoms of patients and timely deal with. If fatal embolism occurs, the consequences are unimaginable. However, performing a thrombectomy presents significant challenges. Firstly, achieving adequate exposure is difficult. While removal of the mitral valve can increase the visual field, it increase the risk of damaging mitral valve. Secondly, the thrombus in trabeculae carneae of the ventricles can't be completely removed, leaving residual thrombi could subsequently enter the systemic or pulmonary circulation once ventricular contractions resume. Lastly, the thrombus may strongly adherence to the endocardium, leading to residual roughened areas following the procedure. This condition further predisposes the patient to thrombosis. So it's a very difficult choice whether or not to surgical thrombectomy.

In the absence of extensive literature and guidelines, each step we undertake is approached with caution and complexity. Although we have successfully used rivaroxaban to dissolve ventricular thrombus, many clinicians are concerned that rivaroxaban cannot be routinely monitored to assess whether therapeutic effect has been achieved, especially in places with poor medical conditions. Same with other non-vitamin-K-antagonist oral anticoagulants. For the current literature on giant biventricular thrombus, all of them are successful in thrombolysis, and there are few data on serious complications or failures in the treatment process, which seriously affects the formulation of current guidelines and consensus. In addition, thrombus and ventricular contractility interact with each other, so whether myocardial contractility can be further improved after the removal of thrombus, the surgical thrombectomy is not getting more support.

## Conclusion

This case demonstrates that aggressive anticoagulation and GDMT can resolve massive biventricular thrombi, even in the presence of cerebral hemorrhage. Key lessons include: (1) Serial imaging guides dose titration; (2) Multidisciplinary input balances thromboembolic and bleeding risks; (3) High-altitude residency necessitates hemoglobin monitoring. Future studies should compare rivaroxaban vs. warfarin in similar cohorts.

## Data Availability

The original contributions presented in the study are included in the article/Supplementary Material, further inquiries can be directed to the corresponding author.
